# The Effect of Ageing on the Mechanical and Tribological Properties of Al-Zn-Mg Alloy

**DOI:** 10.3390/ma19010104

**Published:** 2025-12-27

**Authors:** Jakub Papież, Kacper Leśny, Martyna Zemlik

**Affiliations:** Department of Vehicle Engineering, Faculty of Mechanical Engineering, Wrocław University of Science and Technology, Wybrzeże Wyspiańskiego 27, 50-370 Wrocław, Poland; kacper.lesny@pwr.edu.pl

**Keywords:** aluminium alloy, abrasive wear, impact strength, heat treatment, 7075

## Abstract

The aim of this study was to evaluate the effect of heat treatment, including solutionising and ageing in the temperature range of 20–250 °C, on the microstructural, mechanical, and tribological properties of the Al 7075 alloy. Microscopic analysis revealed that in the as-received condition and after natural ageing, the microstructure is characterised by the presence of elongated grains and a banded distribution of precipitates, whereas higher ageing temperatures lead to their coarsening and the initiation of recrystallisation processes. The highest hardness (189 HV) was obtained after ageing at 100 °C for 48 h, while further increases in temperature caused a systematic decrease in hardness—down to 85 HV at 250 °C for 4 h. Impact tests showed that in the as-received condition, the material reached a value of 7 J/cm^2^, after natural ageing 15 J/cm^2^, and the maximum (26 J/cm^2^) was achieved for samples aged at 250 °C for 4 h. Tribological tests conducted using the T-07 method confirmed the dependence of wear resistance on heat treatment parameters—the lowest relative abrasive wear resistance coefficient was observed after natural ageing (*k_b_* = 0.860), and the highest after ageing at 250 °C for 4 h (*k_b_* = 1.216). The results obtained indicate that moderate ageing conditions (100–150 °C) favour increased hardness, whereas higher temperatures (200–250 °C) lead to an improvement in impact strength and tribological resistance, which showed an inversely proportional relationship with hardness, contrary to Archard’s law.

## 1. Introduction

A breakthrough moment in the history of the light metal industry occurred in 1886, when the electrolytic process for aluminium production was developed. This made it possible to produce aluminium on an industrial scale, significantly reducing production costs and enabling its widespread use. By the end of the 19th century, aluminium had already found applications in, among others, the manufacture of railway carriages, automotive bodies, and electrical power transmission lines. Since then, aluminium and its alloys have become some of the most important structural materials in modern industry [1]. Aluminium is characterised by a very low density (approx. 2.70 g/cm^3^), combined with a high specific strength (for structural alloys up to 300–600 MPa) and chemical resistance, as well as good thermal (approx. 235 W/m·K) and electrical conductivity (approx. 35–38 MS/m) [2]. These properties, combined with its relatively low price, provide wide possibilities for the material’s application. Aluminium is used, among others, in the food industry, for the construction of chemical equipment, heat exchangers, electrical conductors, lightweight structures, automotive rims, engine components (e.g., pistons), energy turbines, and in the production of windows and doors [3,4,5,6].

Aluminium alloys are divided into two main groups: casting alloys and wrought alloys. The latter include the 7xxx series, also known as Al-Zn alloys, which are designed for further heat treatment. Alloys from the 7xxx group belong to the family of heat-treatable materials. They are characterised by a high content of alloying elements, although still below the eutectic concentration. They exhibit the highest strength and fatigue properties among all aluminium alloys, which makes them suitable for the construction of machine and vehicle components, railway rolling stock, and, above all, aerospace structures [7,8].

Aluminium does not undergo phase transformation, which significantly limits the range of applicable processes during heat treatment. Therefore, the key aspect in designing the treatment process is the appropriate selection of time and temperature. Aluminium heat treatment can be divided into two main stages, both aimed at precipitation hardening. The first process is solution heat treatment, which involves heating the material to a temperature that allows the dissolution of intermetallic phases and homogenisation of the chemical composition throughout the volume of the material, followed by rapid cooling, resulting in a supersaturated α solid solution. The next stage is ageing, which can be divided into two types: natural and artificial.

The properties of 7xxx-series alloys are determined by precipitation hardening caused by the formation of solid phases composed mainly of alloying elements [9]. The compounds formed from aluminium, zinc, magnesium, and copper provide very high hardness and strength while maintaining good ductility. Among the phases influencing the strengthening of 7xxx-series aluminium alloys are the Guinier–Preston (GP) zones, which form thin, metastable, and coherent plates with the matrix. These zones act as nuclei for other phases. The GP1 zones form during natural ageing at temperatures up to about 150 °C and with a Zn:Mg ratio close to 1:1, while GP2 zones form during artificial ageing above 70 °C. The GP2 zones are characterised by a higher zinc content compared to magnesium (Zn > Mg) and serve as the basis for the nucleation of the η′ phase. The η′ phase is metastable and semi-coherent with the matrix, has a hexagonal structure, and is the main source of strengthening due to the elastic stresses it induces in the matrix. With further evolution, it transforms into the incoherent η phase. This phase belongs to the Laves phases with a hexagonal C14 structure, and its precipitates mainly act as geometrical obstacles to dislocation movement (Orowan mechanism), thus providing less strengthening than the η′ phase. Generally, the precipitation sequence is considered to follow solid solution (α) → GP zone → η′ (Zn_2_Mg) → η (Zn_2_Mg) [10,11]. Other strengthening phases in the 7xxx-series aluminium alloys include T′ and T (Al_2_Mg_3_Zn_3_ or Al_49_Zn_49_Mg_32_), which form at lower zinc contents, as well as S′ and S (Al_2_CuMg) phases for alloys with higher copper content. Through appropriate heat treatment procedures, the 7075 aluminium alloy achieves the highest mechanical properties within this group—its tensile strength *R_m_* in the T-651 condition reaches 572 MPa, yield strength *R_e_*—503 MPa, elongation A—11%, and hardness—175 HV [12]. Given that the tensile strength *R_m_* of pure aluminium is typically between 70–120 MPa, these values are up to eight times higher. They are also more than twice those of aluminium–manganese alloys (3xxx series), which, after strain hardening, reach an *R_m_* of about 245 MPa [1].

The 7xxx-series aluminium alloys (e.g., 7075, 7475, 7050) perform best under high mechanical loads at moderate temperatures (<150 °C) in corrosion-protected environments. They are sensitive to stress corrosion; therefore, in aerospace applications, their surfaces are protected with coatings [13]. These alloys are used in components exposed to adhesive wear, such as bushings, pins, joints, flap guides, and landing gear mechanisms. They also need to exhibit resistance to abrasive wear, which can occur when sand, dust, or ice particles enter between sliding surfaces—this applies, for instance, to guide components, landing gear struts (especially in light aircraft), as well as flap and slat mechanisms. In the presence of moisture or salt, the environment becomes corrosive-abrasive, and anodising is often applied. However, a soft substrate limits the mechanical properties of the entire component; thus, the matrix should also exhibit satisfactory strength.

It should also be noted that abrasive wear tests are an important method for verifying the mechanical properties of materials, including aluminium alloys, as they allow the assessment of mass loss, surface durability, and suitability for operation under frictional conditions [14]. Therefore, the authors undertook an analysis of the tribological and mechanical properties of heat-treated aluminium alloy 7075 under dry friction conditions using corundum as the abrasive medium. Corundum is particularly useful in tribological tests because its hardness (9 on the Mohs scale) is significantly higher than that of many other abrasive media, such as silica (hardness of 7 on the Mohs scale), allowing for a more demanding and reliable assessment of the wear resistance of the tested materials.

## 2. Materials and Methods

The material used in the study was the EN AW-7075 aluminium alloy, belonging to the group of heat-treatable alloys, characterised by an increased magnesium and zinc content. The chemical composition of the EN AW-7075 core alloy is presented in Table 1. The content of individual elements complies with the requirements of the [15]. Chemical analysis was carried out on the cross-section of a sample by means of a GDS500A Leco glow discharge atomic emission spectrometer (LECO Corporation, St. Joseph, MI, USA) with the following parameters: *U* = 1250 V, *I* = 45 mA, 99.999% argon. The results were the arithmetic averages of five measurements.

The heat treatment was performed in a Czylok FCF 12SHM/R gas-tight chamber furnace (Czylok, Jastrzębie-Zdrój, Poland) under an argon atmosphere (99.95%). The solution-treatment temperature was 480 °C for 1 h—selected to stabilise the properties of the supersaturated alloy. Extending the soaking time did not change the hardness level obtained, which remained 128 HV in each case. The samples were then aged at 100–250 °C in 50 °C increments. The as-received material and the naturally aged alloy at room temperature were used as reference conditions. The parameters, developed based on literature sources [16,17,18,19], are listed in Table 2. The ageing times differ due to the increasing intensity of microstructural transformations at higher temperatures; therefore, similar properties can be achieved for shorter times at higher temperatures or for longer times at lower ones.

Macro- and microscopic examinations were performed respectively with the use of a Nikon AZ100 stereoscopic microscope and a Nikon Eclipse MA200 light microscope (Nikon Corporation, Tokyo, Japan) at magnifications between 25× and 500×. The specimens were etched with a solution of 10% HF. Images were recorded with a Nikon DS-Fi2 digital camera and analysed using NIS Elements software (Photodocumentation package). (https://www.microscope.healthcare.nikon.com/en_EU/products/software/nis-elements/nis-elements-documentation, accessed on 15 November 2024). Images of fractographies and surfaces subjected to wear testing were taken with a Phenom XL electron microscope (Thermo Fisher Scientific, Waltham, MA, USA) using SE imaging and a 15 keV accelerating voltage. The grain size was measured according to [20] using ImageJ ver. 1.52a software.

Hardness was measured using the Vickers method (Matsuzawa MMT-X7b micro-hardness tester, Matsuzawa Co., Akita, Japan) in accordance with [21], with an applied load of 9.807 N.

Impact tests were carried out at room temperature using a Zwick Roell RPK300 Charpy hammer with an initial energy of 300 J (ZwickRoell, Ulm, Germany), in compliance with [22], on full-size V-notched specimens.

**Figure 1:**
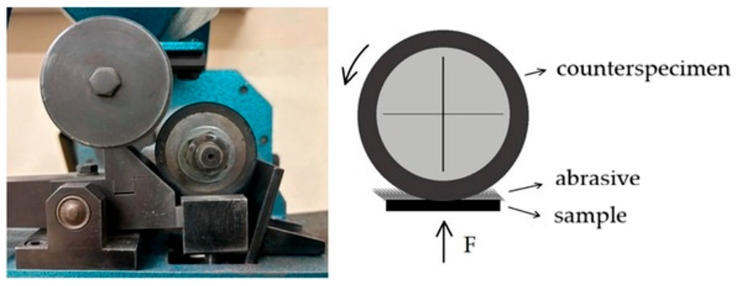
View and layout of the T-07 tribotester [26].

Tribological studies were carried out using the T-07 loose abrasive wear resistance tester (Łukasiewicz—Institute for Sustainable Technologies, Radom, Poland), in accordance with [23]. Each test was performed in six repetitions. The difference between the T-07 tester and the tribotester described in the [24] international standard consists of locating the examined material. For the T-07 tester, the specimen is placed horizontally, and for the tribotester described in ASTM—vertically. The T-07 tribotester consists of a rubber-rimmed steel wheel with a diameter of Ø = 50 (±0.2) mm and a width of 15 (±0.1) mm, an abrasive reservoir, and a counter lever with weights that hold the sample and generate a vertical force to press the sample against the roller (Figure 1). The hardness of the rubber lies in the range of 78–85 ShA. The tests were carried out under a constant load of F = 44 N (∆F = 0.25 N). Electrocorundum with a particle size of #90 was used as an abrasive, according to the Polish standard [25]. The duration of the test depends on the hardness of the test material and was 10 min (600 rotary cycles). The dimensions of the samples were 30 mm × 30 mm × 3 mm. The coefficient of relative abrasion resistance *k_b_*, calculated according to Formula (1), was used as a measure of abrasion resistance. The tests were conducted at a room temperature of 23 °C.

(1) $$k_{b}=\frac{Z_{ww}\rho_{b}N_{b}}{Z_{wb}\rho_{w}N_{w}}$$

where

*k_b_*—coefficient of relative abrasion resistance [dimensionless],
*Z_ww_*—mass consumption of the standard sample [g],
*Z_wb_*—mass consumption of the tested sample [g],
*N_w_*—number of rotations of the rubber-rimmed steel wheel during the test of the standard sample,
*N_b_*—number of rotations of the rubber-rimmed steel wheel during the test of the tested sample,
*ρ_w_*, *ρ_b_*—material density of the standard sample and tested sample [g/cm^3^].

## 3. Results

### 3.1. Materials Analysis

**Figure 2:**
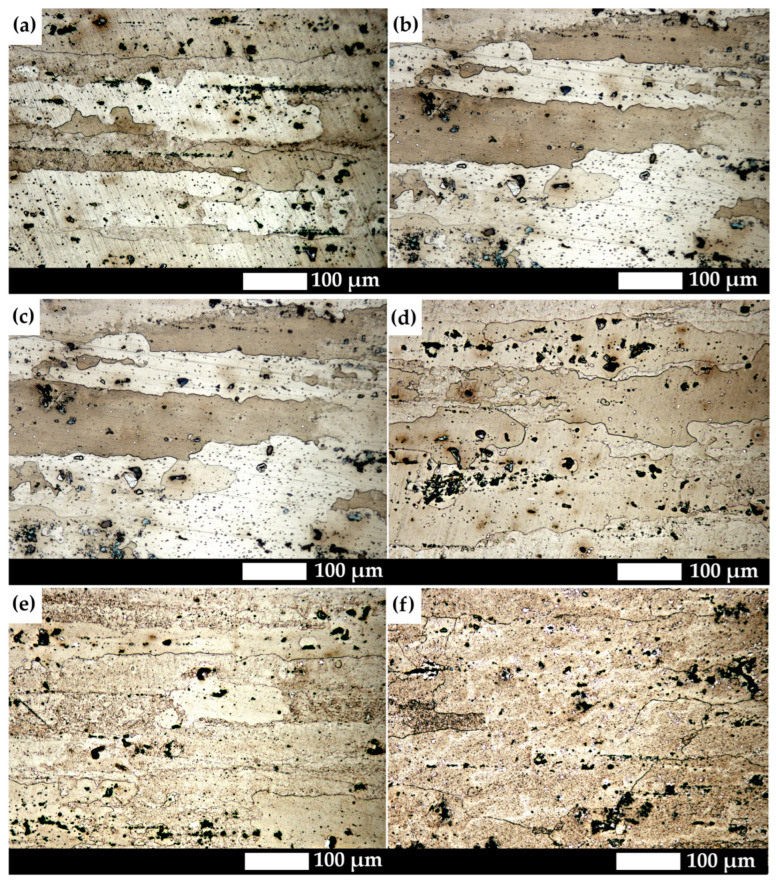
Microstructure of the analysed aluminium alloy: (**a**) as-delivered; (**b**) naturally aged; (**c**) 100 °C/48 h; (**d**) 150 °C/12 h; (**e**) 200 °C/6 h; (**f**) 250 °C/4 h. LM, etched with 10% HF.

Figure 2 presents the microstructures of the analysed aluminium alloy subjected to different heat treatment variants. In the as-delivered state and after natural ageing, the microstructure is characterised by banded impurities and elongated grains of the solid solution, resulting from prior plastic deformation. Secondary-phase precipitates in these conditions are very fine, evenly distributed within the grains, and poorly visible under light microscopy. With increasing ageing temperature (100–150 °C), the precipitates become more distinct, and the grains gradually lose their elongated shape, adopting a more isotropic, oval morphology. After ageing at 200 °C, diffusion processes lead to further coagulation of precipitates, reducing the effect of precipitation strengthening. The most advanced microstructural changes occur after heat treatment at 250 °C, where the initiation of recrystallisation can be observed, manifested by the appearance of more regular, rounded grains and the almost complete disappearance of the banded texture. The precipitates become large and irregular, indicating over-ageing, which accounts for the decrease in mechanical properties.

**Figure 3:**
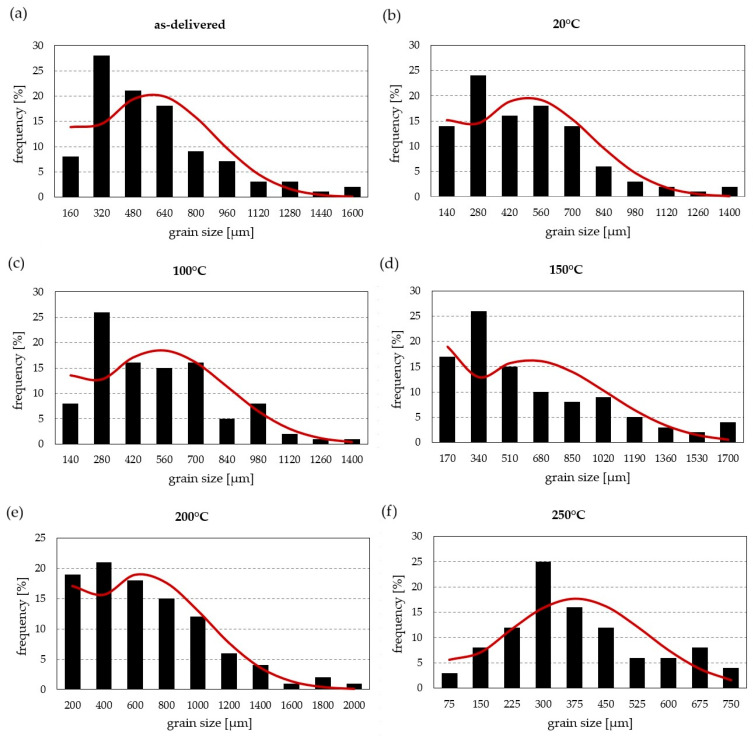
Frequency intervals and normal distributions (red lines) of grain-size occurrence: (**a**) as-delivered; (**b**) natural ageing; (**c**) 100 °C/48 h; (**d**) 150 °C/12 h; (**e**) 200 °C/6 h; (**f**) 250 °C/4 h.

**Figure 4:**
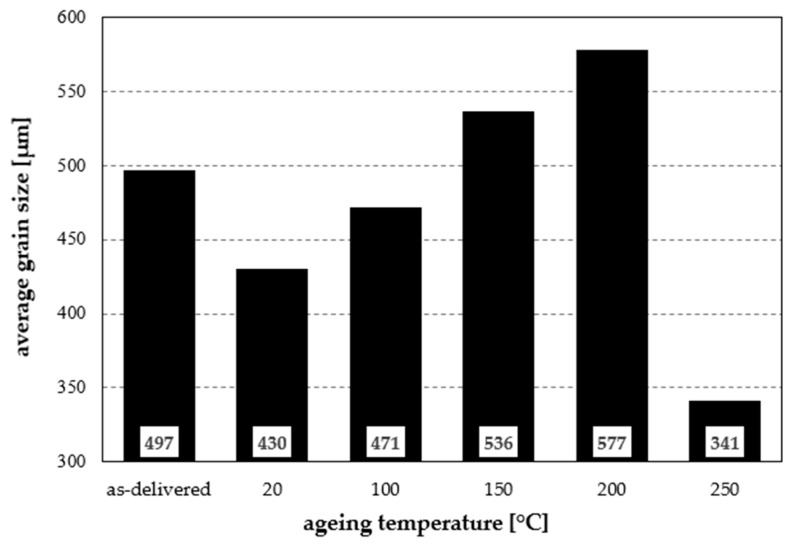
Average grain size of the analysed aluminium alloy subjected to ageing treatments.

These observations are confirmed by the grain-size analysis (Figure 3). In the as-delivered condition, characterised by elongated directional grains, the average grain size is 497 µm (Figure 4). The most numerous grains are in the 160–320 µm range, but individual grains exceeding 1440 µm shift the peak of the distribution to 480–640 µm. Material subjected to solution treatment and natural ageing shows a modified grain-size distribution: the largest grains reach 1400 µm, with a higher share of grains <140 µm and a distribution peak at 420–560 µm. Owing to recrystallisation, the average grain size decreases by 67 µm compared with the as-delivered state, reaching 430 µm. Heat treatment involving solutioning and ageing at 100 °C increases the average grain size to 471 µm, still lower than in the as-delivered condition. The grains are relatively uniform in size, and the share of grains between 280 and 700 µm (for histogram bins of 140 µm) is approximately balanced. Ageing at 150 °C increases the fraction of smaller grains (<170 µm), accompanied by grain growth up to 1700 µm, related to the reduction in grain-boundary density. The mean grain size then increases to 536 µm, exceeding the as-delivered value. A similar effect is observed after heating at 200 °C: most grains are <200 µm, though a few reach up to 2000 µm. Ageing at 250 °C completely changes the grain-size distribution—the most numerous grains are in the 220–300 µm range, the maximum occurs between 300 and 450 µm, and the distribution is nearly symmetrical. The average grain size is the smallest of all analysed conditions and amounts to 371 µm.

**Figure 5:**
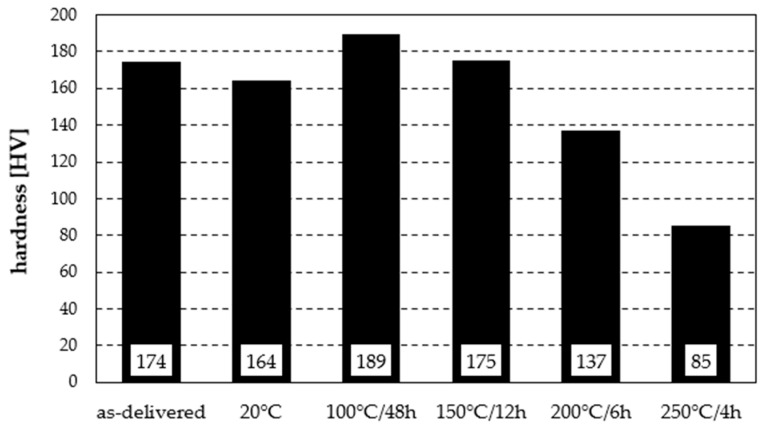
Hardness of the analysed aluminium alloy subjected to various heat treatment variants.

**Figure 6:**
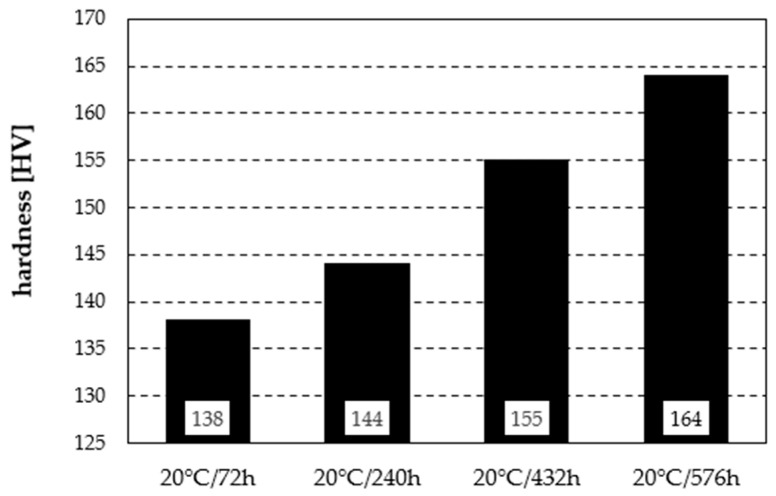
Hardness as a function of time for naturally aged material at 20 °C.

The hardness tests revealed distinct changes in mechanical properties depending on the heat treatment parameters (Figure 5). In the as-delivered state, the hardness was 174 HV. After solution treatment, a decrease to 128 HV was observed, followed by a systematic increase through natural ageing at 20 °C—from 138 HV after 72 h to 164 HV after 576 h (Figure 6). In the case of artificial ageing, the hardness remained stable regardless of the time intervals between measurements. Treatment at 100 °C for 48 h resulted in the highest hardness in the series—189 HV. Ageing at 150 °C/12 h caused a drop to 175 HV, comparable to the as-delivered condition. Further temperature increases led to a clear decline: 137 HV at 200 °C/6 h and 85 HV at 250 °C/4 h. It should also be noted that due to the high repeatability of the results—and consequently the low standard deviation (of at most 2 Vickers hardness units)—the authors refrained from performing a statistical interpretation of the test data.

Thus, moderate ageing conditions (100 °C) provided the greatest strengthening of the alloy, whereas higher temperatures systematically reduced hardness due to precipitate coarsening and growth. Natural ageing at 20 °C showed a slower but steady increase, approaching the initial hardness value.

### 3.2. Impact Strength and Fractographic Analysis

**Figure 7:**
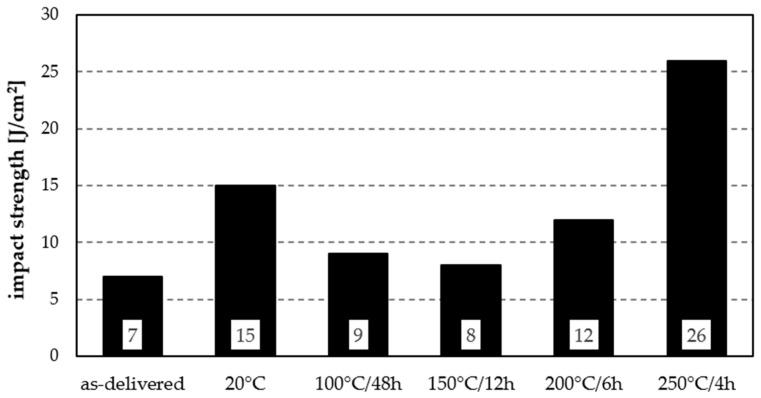
Impact strength of the analysed aluminium alloy subjected to various heat treatment variants.

Impact testing of the Al7075 alloy subjected to different ageing variants showed significant changes in fracture resistance, depending on heat treatment parameters (Figure 7). In the initial state, the impact energy was 7 J/cm^2^. Natural ageing at 20 °C more than doubled it to 15 J/cm^2^. Artificial ageing resulted in lower values: 9 J/cm^2^ (100 °C/48 h), 8 J/cm^2^ (150 °C/12 h), and 12 J/cm^2^ (200 °C/6 h). The highest impact strength, 26 J/cm^2^, was obtained after ageing at 250 °C/4 h—nearly a fourfold increase relative to the as-delivered condition.

In summary, moderate ageing conditions (100–150 °C) did not enhance impact strength, while higher temperatures (200–250 °C) produced a marked increase, with a maximum after 250 °C/4 h treatment. Nevertheless, the values remain insufficient for dynamic-load resistance, since, according to [27], the brittleness threshold is classified at 35 J/cm^2^.

**Figure 8:**
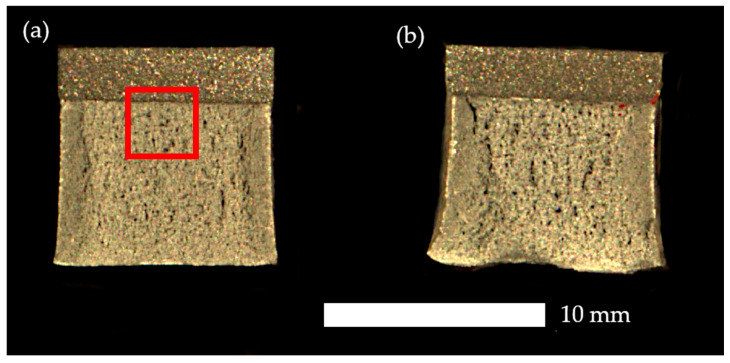
Fracture surfaces in the under-notch region of the analysed aluminium alloy: (**a**) natural ageing; (**b**) ageing at 250 °C/4 h. The area marked in red indicates the region where more detailed microscopic observations were performed.

Additional information regarding fracture behaviour was obtained from the analysis of fractographic surfaces. Figure 8 presents two representative conditions: naturally aged material (a) and material aged at 250 °C (b). In the first case, the fracture surface is relatively flat, which is typical for a brittle fracture mechanism. Distinct parallel steps are visible across the entire surface, indicating directional crack propagation and the limited ability of the material to absorb impact energy. Locally, small dimples can be observed associated with the presence of impurities that initiated the cracking process.

In the case of the material aged at 250 °C, parallel steps are also visible; however, the fracture surface is more irregular. In the final breaking zone, rounded (plastically deformed) edges are visible, which indicates partial energy absorption before failure. Moreover, in Figure 8a, the region below the notch is marked, where more detailed microscopic observations were performed to define the fracture characteristics for all analysed heat treatment variants.

**Figure 9:**
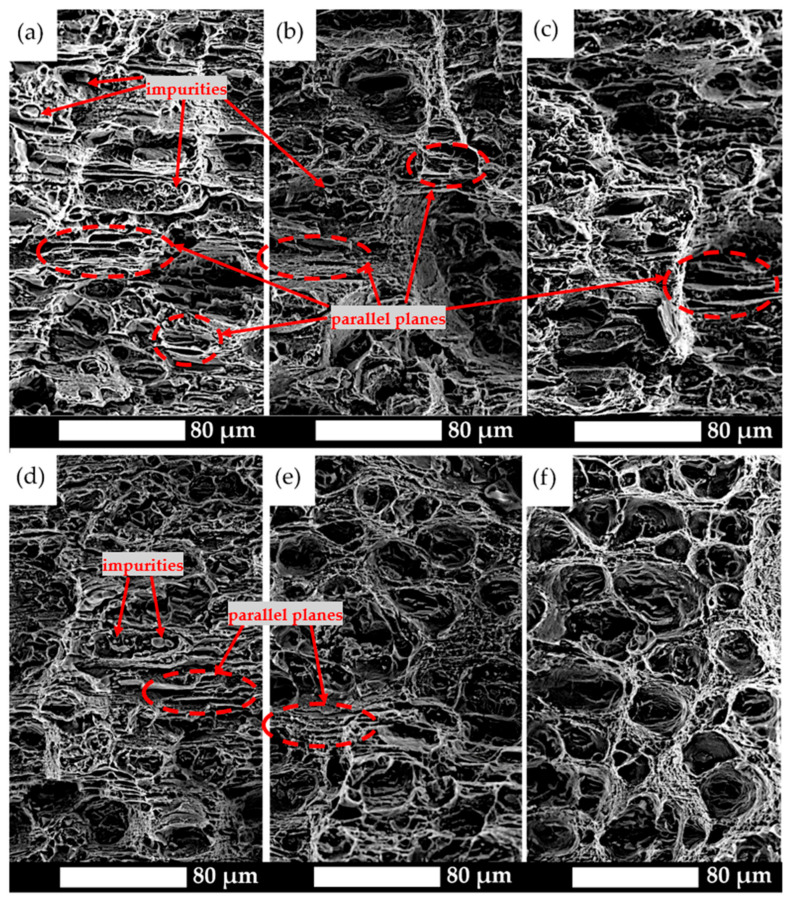
Fractographic images of the fracture surfaces in the under-notch region of the analysed aluminium alloy: (**a**) as-delivered; (**b**) natural ageing; (**c**) 100 °C/48 h; (**d**) 150 °C/12 h; (**e**) 200 °C/6 h; (**f**) 250 °C/4 h. SEM, unetched.

SEM images of the fracture surfaces in the under-notch region, where tensile stresses act (marked in red in Figure 8), revealed that in the as-delivered condition, naturally aged material, and after ageing at 100 °C and 150 °C, the fracture surface was dominated by elongated, parallel planes along which decohesion occurred (Figure 9). At their edges, no plastic tearing was observed, and the boundaries were sharply terminated, indicating the joining of parallel cleavage planes within the grain. Only locally were dimples present, at the bottoms of which impurities could be seen, serving as crack initiation sites.

With increasing ageing temperature, the share of ductile fracture areas increased. The presence of characteristic parabolic dimples and elongated edges indicates a change in the fracture mechanism. Stretched ridges, which make it impossible to identify cleavage planes, are typical for material aged at 150 °C and 200 °C, where the fracture should be classified as quasi-cleavage. Material subjected to ageing at 250 °C exhibits feature characteristics of ductile fracture with a fibrous structure, as evidenced by slightly elongated dimples and inclusions visible at the bottoms of the largest ones.

### 3.3. Abrasive Wear Resistance Tests

**Figure 10:**
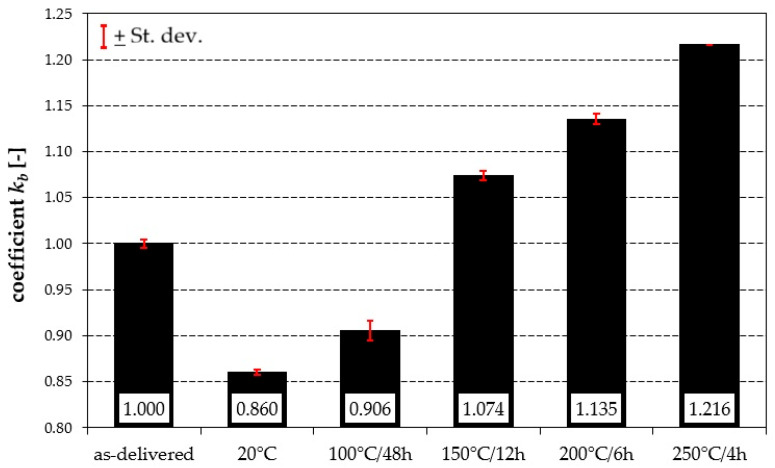
Relative abrasion resistance indices of the analysed aluminium alloy subjected to various heat treatment variants.

Figure 10 shows the values of the coefficient of relative abrasion resistance *k_b_* for the Al7075 aluminium alloy subjected to various heat treatment variants, together with the corresponding standard deviations. It should be noted that in the as-delivered condition, the alloy exhibited a *k_b_* value of 1.00 ± 0.004, which was adopted as the reference point for further analysis. Tribological tests showed that the lowest value of the coefficient *k_b_* was obtained for samples aged at 20 °C (*k_b_* = 0.860 ± 0.003), indicating the lowest resistance to abrasive wear in this condition. A slightly higher value was recorded after ageing at 100 °C/48 h (*k_b_* = 0.906 ± 0.011). With increasing ageing temperature and duration, a systematic rise in the coefficient *k_b_* was observed—up to 1.074 ± 0.005 for 150 °C/12 h and 1.135 ± 0.006 for 200 °C/6 h. The highest resistance to abrasive wear was found for samples aged at 250 °C/4 h, where *k_b_* reached a value of 1.216, and the results obtained were completely repeatable, as confirmed by the absence of standard deviation.

The results clearly demonstrate a distinct relationship between heat treatment parameters and tribological resistance, indicating that more intensive ageing conditions promote higher resistance to the abrasive wear of the Al7075 alloy. The low standard deviation values (0.000–0.011) confirm the good repeatability and homogeneity of the experimental results.

**Figure 11:**
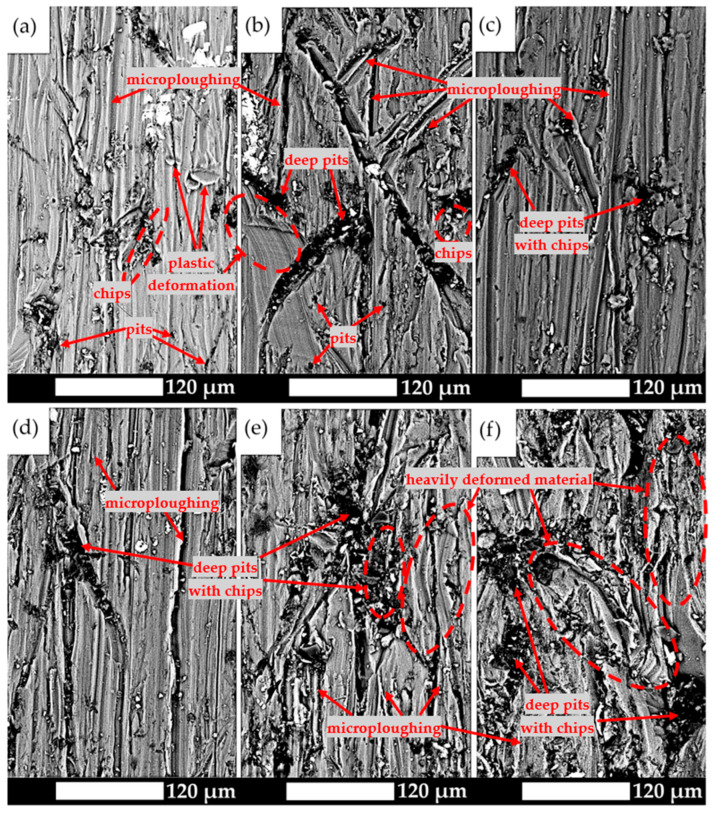
Images of the worn surfaces of the analysed aluminium alloy subjected to testing with the use of the T-07 device: (**a**) as-delivered; (**b**) natural ageing; (**c**) 100 °C/48 h; (**d**) 150 °C/12 h; (**e**) 200 °C/6 h; (**f**) 250 °C/4 h. SEM, unetched.

The above results are consistent with microscopic observations of the surfaces subjected to wear tests (Figure 11). It should be noted that for the as-delivered material, the dominant wear mechanism is microploughing. Numerous continuous chips are also observed, resulting from the high plasticity of the matrix, which prevents their easy detachment and complicates their removal from the friction zone (Figure 11a). The grooves formed are relatively narrow, and their formation is associated with plastic deformation and work hardening of the material, which leads to the initiation of microcracks and their subsequent widening. The resulting scratches are approximately parallel to the direction of abrasive particle motion and only locally show deviations in orientation. It should also be emphasised that the obtained microscopic images confirm the plastic nature of the material despite its low impact toughness, which should be attributed to the strengthening effect of secondary phases while maintaining a deformable matrix.

A clear change in the wear character is observed for the naturally aged material (Figure 11b). The pits are distinctly wider and deeper, although grooves and chips are still visible at their edges. A higher number of small pits appear, resulting from the presence of a soft matrix, which is the first to wear due to microploughing. As the wear process progresses, the softer phase undergoes plastic deformation and abrasion, exposing areas with harder phases. Over time, the harder components begin to protrude above the surface, acting as a natural barrier protecting the matrix from further wear. However, once a certain height is reached, the abrasive particles can also detach this phase, leading to further surface damage and intensifying the wear process. For material aged at 100 °C and 150 °C (Figure 11c,d), the wear mechanisms are similar. The scratches are mainly parallel, and only a few chips and grooves are observed at the edges of the pits, indicating limited plasticity of the material. Consequently, in tribological tests based on mass-loss measurements, lower wear rates are recorded.

At higher ageing temperatures, however, wear processes intensify and the surface damage becomes more severe (Figure 11e,f). Wide grooves and deep pits are visible, confirming that material removal occurs through scratching, microcutting, or fatigue wear caused by repeated plastic deformation of the subsurface layers. Within the pits, tangled chips can also be seen, which do not contribute to a reduction in mass loss. Therefore, it should be concluded that with increasing heat treatment temperature, the hardness of the alloy decreases, which results in a change in the dominant wear mechanism to microploughing—a process that does not significantly reduce the total mass loss.

## 4. Discussion

**Figure 12:**
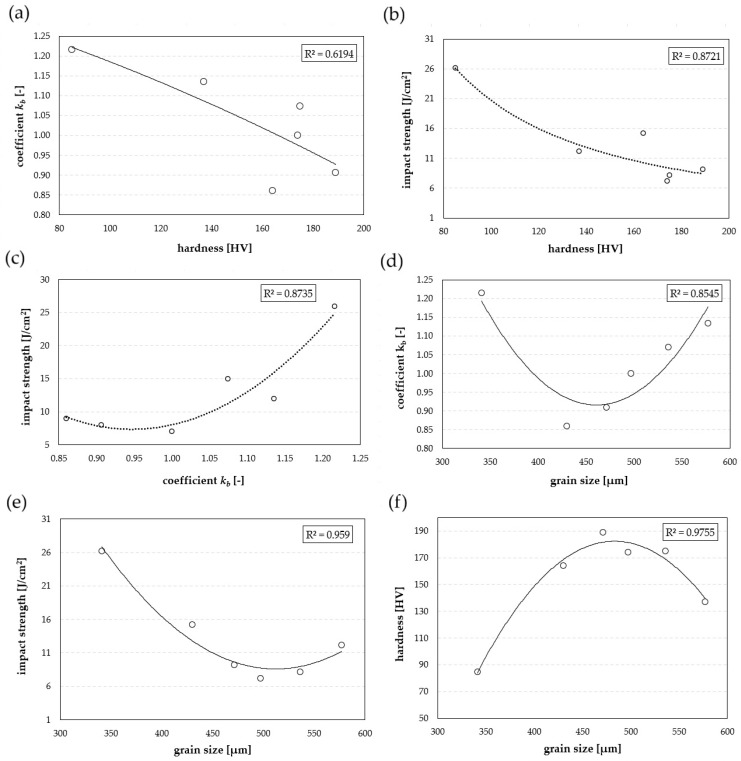
Mutual correlations between abrasive wear resistance, microstructural grain size, hardness, and impact strength: (**a**) coefficient *k_a_* vs. hardness, (**b**) impact strength vs. hardness, (**c**) impact strength vs. coefficient *k_a_*, (**d**) coefficient *k_a_* vs. grain size, (**e**) impact strength vs. grain size, and (**f**) hardness vs. grain size. The solid curves represent the fitted trend lines.

In this study, the microstructural, mechanical, and tribological properties of the 7075 aluminium alloy after heat treatment were determined. Analysis of the relationships between hardness, impact strength, the *k_b_* coefficient, and grain size (Figure 12a–f) revealed significant regularities. According to Figure 12a, a distinct decrease in the *k_b_* coefficient is associated with an increase in hardness. The coefficient of determination R^2^ = 0.6194 indicates a moderate but still noticeable correlation between these parameters, with an inversely proportional relationship. This means that more strongly hardened samples exhibit less favourable tribological performance, which contradicts the classical Archard model, according to which higher material hardness should lead to reduced intensity of abrasive wear [28].

Available literature data have indicated a clear correlation between increasing Brinell hardness, tensile strength, and lower wear in aluminium alloys containing interdendritic precipitates of MgZn_2_ and Al_2_Mg_3_Zn_3_ phases [29]. In study [30], the highest wear of AA7075 alloy was observed sequentially for non-aged samples and those aged at 110, 130, and 120 °C. Using the pin-on-disk method, it was demonstrated that higher hardness corresponded to lower wear. In study [31], it was shown that the Al-Zn-Mg alloy after ageing exhibited hardness in the range of 165–227 HV1. The lowest hardness (165 ± 9.48 HV1) was obtained after ageing at 190 °C for 9 h and corresponded to the greatest wear, whereas the highest hardness (227 ± 9.57 HV1) was recorded for samples aged at 130 °C for 5 h, which resulted in the lowest wear. The applied method involved pressing the samples against a rotating disk covered with abrasive paper under a constant force of 4.91 N. Similar results were obtained by the authors of study [32], who examined six aluminium alloys (AA1050, AA2014-T6, AA3003, AA5052, AA6061-T6, and AA6351-T6) using the dry sand rubber wheel method. In that case, a linear increase in wear with load and a correlation of wear intensity with hardness, tensile strength, and ductility were also found, in agreement with Archard’s law.

At the same time, it should be emphasised that some authors have indicated the limited usefulness of hardness as the sole criterion for predicting resistance to abrasive wear. Studies of an Al-Si1MgMn alloy reinforced with Al_2_O_3_ particles showed that under mild abrasive conditions, the reinforcement improved wear resistance, whereas under higher loads and with harder abrasive grains, this effect disappeared, and wear was even higher than in the unreinforced material [33]. An analogous inverse linear relationship was found for 5xxx-series alloys, where cold-work-hardened material exhibited greater mass loss than the softer condition [34]. For 7xxx-series alloys, less favourable wear indices were observed in the naturally aged condition compared with artificially aged material, which is also confirmed by the results presented in this study. In [14], it was reported that the 7xxx alloy in the T73 condition—despite approximately 10% lower hardness and strength compared with the T651 condition—exhibited the best tribological resistance (an increase in *k_b_* by 22% and a reduction in mass loss by 18%). These findings confirm that the plasticity of the material plays a crucial role in the wear mechanism, as it allows for more effective dissipation of mechanical energy in the contact zone with abrasive particles, delaying crack initiation and propagation [26].

Therefore, the Archard wear equation was applied to the present dataset in order to verify whether hardness alone can be used to predict mass loss under three-body abrasive conditions (Equation (2)). For each ageing state, the volumetric wear loss was obtained from the measured mass loss using a density of 2800 kg/m^3^, and the corresponding hardness was converted from HV to SI units (H = HV × 9.807 × 10^6^ N/m^2^). Individual wear coefficients k calculated from Archard’s relation ranged from approximately 0.0026 to 0.0077, with an average value of k = 0.00571. This mean coefficient was then used to predict the volumetric and mass losses as a function of hardness for all ageing conditions.

(2) $$\;I_{z}=\frac{k\times P\times l}{H}$$

where

*I_Z_*—the volumetric wear loss (m^3^),
*k*—the wear coefficient,
*P*—the normal load (N),
*l*—the sliding distance (m),
*H*—the hardness of the softest contacting surfaces (N/m^2^).

The comparison between experimentally measured and analytically predicted mass loss shows only moderate agreement at higher hardness levels (174–189 HV), where the relative error of the Archard model remains in the range of about 5 to −26% (Table 3). However, for the softer states (137 and 85 HV), the model substantially overestimates wear, with relative deviations reaching approximately +28% and +121%, respectively. These results indicate that, under the present dry sand rubber wheel conditions, hardness alone is not a sufficient predictor of abrasive wear resistance. The deviation from Archard’s law becomes particularly pronounced in the over-aged conditions, where plastic deformation, microploughing, and the formation of mechanically mixed and tribo-oxidised layers dominate the wear process, allowing softer but more ductile microstructures to dissipate energy more effectively and thus to exhibit lower mass loss than would be expected from hardness-based models.

A strong correlation was found between impact strength, hardness, the *k_b_* coefficient, and microstructural grain size (Figure 12b,c). The high determination coefficients (R^2^ > 0.87) confirm a significant correlation between the analysed parameters, according to which an increase in impact strength is associated with a decrease in hardness (Figure 12b) and a reduction in mass loss (Figure 12c). In each of the above cases, the dependence was described by a second-degree polynomial function; however, its deviation was most pronounced in the case of tribological resistance as a function of grain size, indicating a positive relationship only up to a certain level—beyond which further grain growth may also result in increased resistance. It should be emphasised that the microstructure with the largest average grain size is characterised by the presence of coagulated precipitates and a soft matrix, which promotes the ploughing wear mechanism. Therefore, it can be concluded that resistance to abrasive wear is closely related to the material’s ability to accommodate large plastic deformations. Consequently, a fine-grained microstructure combined with a high impact strength contributes to a reduction in wear intensity. The obtained determination coefficients (R^2^ = 0.8721 and R^2^ = 0.8735) clearly indicate that impact strength and grain size constitute more reliable criteria for predicting tribological resistance than hardness. The observed phenomenon is consistent with the classical Hall–Petch relationship [35,36], according to which grain boundaries act as barriers to dislocation motion, requiring higher stresses to overcome them and promoting fracture-energy absorption. As a result, both the strength (Figure 12f) and impact properties of the alloy improved. The highest impact strength (26 J/cm^2^) was obtained for samples with an average grain size of 341 µm.

In summary, the high degree of fit observed in most analysed cases confirms a strong correlation between structural parameters and mechanical properties. The obtained results enable not only a qualitative but also a quantitative description of the influence of heat treatment on wear mechanisms. They can also serve as a basis for optimising processing parameters aimed at obtaining a fine-grained structure that combines high impact toughness with low wear intensity.

## 5. Conclusions

1. In the as-delivered condition and after natural ageing, the microstructure of the Al 7075 alloy is characterised by banded, elongated grains and fine secondary-phase precipitates. With increasing ageing temperature, coagulation of these precipitates and recrystallisation processes occur, leading to the formation of more isotropic grains.
2. The highest hardness (189 HV) was obtained after ageing at 100 °C/48 h, whereas increasing the temperature above 150 °C caused a systematic decrease in hardness—down to 85 HV after ageing at 250 °C/4 h.
3. Impact tests showed that moderate ageing conditions (100–150 °C) do not improve the material’s resistance to fracture, while higher temperatures lead to a significant increase in impact strength. The highest value—26 J/cm^2^—was obtained after ageing at 250 °C/4 h, representing nearly a fourfold increase compared with the as-delivered condition.
4. The resistance to abrasive wear of the Al 7075 alloy increases with the ageing temperature. The lowest value of the *k_b_* coefficient (0.860) was obtained after natural ageing, and the highest (*k_b_* = 1.216) after heat treatment at 250 °C/4 h.
5. An inverse correlation was found between hardness and resistance to abrasive wear—the samples with the highest hardness did not exhibit the most favourable wear-resistance indices. Impact strength and grain size proved to be more reliable criteria for predicting the tribological performance of the Al 7075 alloy, as confirmed by the high coefficients of determination (R^2^ = 0.8721 and R^2^ = 0.8735).

## Figures and Tables

**Table 1:** Chemical composition of the alloy and content of individual elements in accordance with EN-573:3 2019.

%Al	%Zn	%Mg	%Cu	%Fe	%Si	%Mn	%Cr	%Zr	%Ti	% Others
EN-573:3 2019										
Remainder	5.1–6.1	2.1–2.9	1.2–2.0	Max 0.5	Max 0.4	Max 0.3	0.18–0.28	Max 0.25	Max 0.20	Max 0.05
AA7075										
89.7	5.7	2.74	1.47	0.15	-	0.0039	0.202	-	0.0307	0.0034

**Table 2:** Parameters of the applied heat treatment.

No	Solution Treatment	Ageing	
		Temperature [°C]	Time [h]
1	480 °C/1 h	100	48
2		150	12
3		200	6
4		250	4

**Table 3:** Mass consumption and volumetric wear loss determined experimentally and predicted by the Archard model.

State of Heat Treatment	Actual Mass Consumption (g)	Actual Volumetric Wear Loss I_exp_ (m^3^)	Wear Coefficient k Determined Empirically	Wear Coefficient k Used in the Archard Wear Model	Theoretical Volumetric Wear Loss I_Z_ (m^3^)	Relative Difference (%)
as-delivered	0.131	4.69 × 10^8^	0.00643	0.00571	4.16 × 10^8^	−11.2
20 °C	0.153	5.45 × 10^8^	0.00705		4.42 × 10^8^	−19.0
100 °C	0.145	5.18 × 10^8^	0.00772		3.83 × 10^8^	−26.0
150 °C	0.122	4.37 × 10^8^	0.00603		4.14 × 10^8^	−5.21
200 °C	0.116	4.13 × 10^8^	0.00446		5.29 × 10^8^	28
250 °C	0.108	3.86 × 10^8^	0.00258		8.52 × 10^8^	121

## Data Availability

The original contributions presented in this study are included in the article. Further inquiries can be directed to the corresponding authors.
